# Advanced application of bovine intestinal epithelial cell line for evaluating regulatory effect of lactobacilli against heat-killed enterotoxigenic *Escherichia coli*-mediated inflammation

**DOI:** 10.1186/1471-2180-13-54

**Published:** 2013-03-07

**Authors:** Naoya Takanashi, Yohsuke Tomosada, Julio Villena, Kozue Murata, Takuya Takahashi, Eriko Chiba, Masanori Tohno, Tomoyuki Shimazu, Hisashi Aso, Yoshihito Suda, Shuji Ikegami, Hiroyuki Itoh, Yasushi Kawai, Tadao Saito, Susana Alvarez, Haruki Kitazawa

**Affiliations:** 1 Food and Feed Immunology Group, Laboratory of Animal Products Chemistry, Graduate School of Agricultural Science, Tohoku University, Sendai, Aoba-ku, 981-8555, Japan; 2 Laboratory of Clinical and Experimental Biochemistry, Reference Centre for Lactobacilli (CERELA-CONICET), Tucuman, Argentina; 3 National Agriculture and Food Research Organization, National Institute of Livestock and Grassland Science, Nasushiobara, 329-2793, Japan; 4 Laboratory of Animal Breading and Genetics, Graduate School of Agricultural Science, Tohoku University, Sendai, 981-8555, Japan; 5 Cell Biology Laboratory, Graduate School of Agricultural Science, Tohoku University, Sendai, Aoba-ku, 981-8555, Japan; 6 Department of Food, Agriculture and Environment, Miyagi University, Sendai, 982-0215, Japan; 7 Division of Research and Development, Food Science Institute, Meiji Dairies Co, Kanagawa, Odawara, 250-0862, Japan

**Keywords:** Bovine intestinal epithelial cells, Immunobiotic, ETEC PAMPs, TLRs negative regulators, *Lactobacillus casei* OLL2768

## Abstract

**Background:**

Previously, a bovine intestinal epithelial cell line (BIE cells) was successfully established. This work hypothesized that BIE cells are useful in vitro model system for the study of interactions of microbial- or pathogen-associated molecular patterns (MAMPs or PAMPs) with bovine intestinal epithelial cells and for the selection of immunoregulatory lactic acid bacteria (LAB).

**Results:**

All toll-like receptor (TLR) genes were expressed in BIE cells, being TLR4 one of the most strongly expressed. We demonstrated that heat-stable PAMPs of enterotoxigenic *Escherichia coli* (ETEC) significantly enhanced the production of IL-6, IL-8, IL-1α and MCP-1 in BIE cells by activating both NF-κB and MAPK pathways. We evaluated the capacity of several lactobacilli strains to modulate heat-stable ETEC PAMPs-mediated inflammatory response in BIE cells. Among these strains evaluated, *Lactobacillus casei* OLL2768 attenuated heat-stable ETEC PAMPs-induced pro-inflammatory response by inhibiting NF-κB and p38 signaling pathways in BIE cells. Moreover, *L*. *casei* OLL2768 negatively regulated TLR4 signaling in BIE cells by up-regulating Toll interacting protein (Tollip) and B-cell lymphoma 3-encoded protein (Bcl-3).

**Conclusions:**

BIE cells are suitable for the selection of immunoregulatory LAB and for studying the mechanisms involved in the protective activity of immunobiotics against pathogen-induced inflammatory damage. In addition, we showed that *L*. *casei* OLL2768 functionally modulate the bovine intestinal epithelium by attenuating heat-stable ETEC PAMPs-induced inflammation. Therefore *L*. *casei* OLL2768 is a good candidate for in vivo studying the protective effect of LAB against intestinal inflammatory damage induced by ETEC infection or heat-stable ETEC PAMPs challenge in the bovine host.

## Background

Enterotoxigenic *Escherichia coli* (ETEC) are pathogenic bacteria that are able to infect humans and several species of animals. In farm animals such as cattle, ETEC infection results in reduced growth rate, increased mortality and economic loss [1]. ETEC interacts with intestinal epithelial cells (IECs), colonizes the small intestine and secretes enterotoxins inducing intestinal acute diarrhea and inflammation [2,3]. In addition to its capacity to infect cells and induce damage through toxins, ETEC are able to induce an inflammatory response through other pathogen-associated molecular patterns (PAMPs) such as lipopolysaccharide (LPS) that contribute to cellular and tissue damage during infections [2,4]. ETEC is able to trigger toll-like receptor (TLR)-4 activation and cytokines production by IECs and induce the recruitment and activation of inflammatory cells. Although this mechanism represent an important primary line of host defense, a prolonged or non-regulated pro-inflammatory cytokines production may lead to tissue damage and epithelial barrier disfunction [1,4,5]. Therefore, during ETEC infection it is imperative to generate an adequate inflammatory response against the pathogen, accompanied by efficient regulation, in order to achieve protection without damaging host tissues.

Probiotics have been defined as “live microorganisms which when administered in adequate amounts confer a health benefit on the host” [6]. Several lactic acid bacteria (LAB) strains are considered beneficial to the host and as such have been used as probiotics and included in several functional foods. Modulation of host immunity is one of the most commonly alleged benefits of the consumption of probiotics. The term immunobiotics has been proposed for those probiotic strains with immunoregulatory activities [7]. Studies have shown that immunobiotics can beneficially modulate the immune response against ETEC [8-11]. Roselli *et al.*[8] showed that *Bifidobacterium animalis* MB5 and *Lactobacillus rhamnosus* GG protect intestinal Caco-2 cells from the inflammation-associated response caused by ETEC K88 by partly reducing pathogen adhesion and by counteracting neutrophil migration. Moreover, experiments in Caco-2 cells demonstrated that *L*. *rhamnosus* GG is able to counteract the ETEC-induced up-regulation of interleukin (IL)-1β and tumor necrosis factor (TNF), and the down-regulation of transforming growth factor β1 (TGF-β1) expression, and consequently to block the cytokine deregulation [9]. In addition, comparative studies between *L*. *rhamnosus* GG and *B*. *animalis* MB5, demonstrated that individual strains of probiotics have a different impact on the inflammatory response triggered in IECs [9]. Others studies evaluating the effect of probiotic yeasts showed that *Saccharomyces cerevisiae* CNCM I-3856 decreased the expression of pro-inflammatory mediators IL-6, IL-8, CCL20, CXCL2, CXCL10 in porcine intestinal epithelial IPI-2I cells cultured with F4+ ETEC [10]. Moreover, it was demonstrated that the CNCM I-3856 strain inhibits ETEC-induced expression of pro-inflammatory cytokines and chemokines transcripts and proteins and that this inhibition was associated to a decrease of ERK1/2 and p38 mitogen-activated protein kinases (MAPK) phosphorylation and to an increase of the anti-inflammatory peroxisome proliferator-activated receptor-γmRNA level [11].

There is increasing research in the use of probiotics for decreasing pathogen load and ameliorating gastrointestinal disease symptoms in animals [12-15]. Several studies were conducted in vivo utilizing different probiotic strains to evaluate the effect of immunobiotics against ETEC infection, however the majority of these studies were performed in swine and only few in the cattle [12]. Beside the in vivo studies, several in vitro tests can be performed to identify the best potential probiotics. In this sense, we have recently demonstrated that the combination of the nuclear factor κ B (NF-κB)-reporter assay using a porcine TLR2-expressing transfectant (HEK^pTLR2^ system), the mitogenic assay using porcine Peyer’s patches immunocompetent cells and the evaluation of anti-inflammatory activities of LAB in porcine intestinal epithelial (PIE) cell line are useful tools to select potential probiotic strains [13]. Moreover, we showed that PIE cells can be used to study the mechanisms involved in the protective activity of immunobiotics against intestinal inflammatory damage and may provide useful information for the development of new immunologically functional feeds that help to prevent inflammatory intestinal disorders, including weaning-associated intestinal inflammation in pigs [14,15]. Therefore the use of probiotics in animal feeding could be enhanced by preliminarly in vitro screening such as the production of inhibitory substances, survival in the gastrointestinal tract and antibiotic susceptibility [16] that can be analyzed to evaluate functionality and safety [12]. Moreover, the use of probiotics in cattle could be improved by the development of in vitro systems specific for cattle that allow the simply and efficient assess of the immunomodulatory activity of the potential probiotic LAB strains.

Recently we have successfully established a bovine intestinal epithelial cell line (BIE cells) [17]. We hypothesized that BIE cells are useful in vitro model system for the study of interactions between pathogens and bovine IECs, for the selection of immunobiotic microorganisms and for the study of the mechanisms of immunomodulation by probiotic LAB in IECs. Therefore, the aims of the present study were: i) to assess the capability of BIE cells to respond to the challenge with heat-stable ETEC PAMPs, considering the production of cytokines and the activation of MAPK and NF-κB pathways and; i) to select potential immunomodulatory LAB that may be used to beneficially modulate the inflammatory response in bovine IECs challenged with heat-stable ETEC PAMPs.

## Methods

### BIE cells

The bovine intestinal epithelial cell line (BIE cells) was originally derived from fetal bovine intestinal epitheliocytes, and then established as a SV40 large T antigen-transformed intestinal cell line as previously described [17]. BIE cells were maintained in Dulbecco’s modified Eagle medium (DMEM; GIBCO, Grand Island, NY) containing 10% heat-inactivated fetal bovine serum (FBS) and penicillin-streptomycin until it they were used for further studies. For the passage, BIE cells were treated with a sucrose/EDTA buffer (0.1M Na_2_HPO_4_/12H_2_O, 0.45M Sucrose, 0.36% EDTA/4Na, BSA) for 4 min, detached using 0.04% trypsin in phosphate-buffered saline (PBS, pH7.2) [18]. Then, BIE cells were plated in collagen type I-coated culture dishes (Sumilon, Tokyo, Japan) at 1.5×10^4^ cells/cm^2^ and cultured at 37°C in an atmosphere of 5% CO_2_ in Dulbecco’s Modified Eagle media (DMEM) (10% FCS, 1% streptomycin/penicillin, 100 U/ml streptomycin, high glucose, L-glutamine, 0.11 mg/ml sodium pyruvate; GIBCO).

### Microorganisms

Enterotoxigenic *Escherichia coli* (ETEC) strain 987 (O9: H-: 987P+: STa+) was kindly provided by Dr. M. Nakazawa, National Institute of Animal Health (Tsukuba, Japan) [19]. ETEC cells were grown in blood agar (5% sheep blood) for 24 hours at 37°C and then transferred to tryptic soy broth (TSB; Becton, Dickinson and Company, USA) for 5 days at 37°C without shaking to get a pellicle containing piliated phase. ETEC cells were collected from the pellicle and transferred to 1L TSB and cultured 20 hours at 37°C with shaking. After incubation, the subcultures of bacteria were centrifuged at 5000 × g for 10 min at 4°C and washed with PBS (pH7.2). Finally, ETEC cells were heat killed at 100°C for 15 minutes and then washed with PBS. Heat-stable ETEC PAMPs were suspended in DMEM for use. The following lactobacilli strains were used in this study: *Lactobacillus reuteri* MEP221101 and MEP221102, *Lactobacillus casei* MEP221103, OLL2768, MEP221104, MEP221105, MEP221106, MEP221107, MEP221108, MEP221109, MEP221114 and MEP221115, *Lactobacillus rhamnosus* MEP221110, MEP221111, MEP221112 and GG, *Lactobacillus salivarius* MEP221113, *Lactobacillus jensenii* TL2937 and *Lactobacillus gasseri* MEP221117. The lactobacilli strains were grown in de Man, Rogosa and Sharpe (MRS) medium (Difco, Detroit, USA) for 16 h at 37°C and washed with PBS (pH7.2), and heat killed (60°C, 30 min). These bacterial samples were resuspended in DMEM, enumerated using a Petroff-Hausser counting chamber, and stored at −80°C until use [14].

### Immunocytochemistry

BIE cells were cultured at a cell density of 3×10^4^ cells/well of a 12-well culture plate collagen type I-coated glass disk (Iwaki Glass Co., Tokyo, Japan) for 3 days, (37°C, 5% CO_2_). BIE cells were washed with cold PBS (pH7.2) plus 2% FCS twice and then fixed with 4% paraformaldehyde/PBS solution (room temperature, 5 minutes). Following treating with PBS-T (0.2% Triton X-100) for 5 min at room temperature and washing three times with PBS. Cells were then incubated with Alexa 488 conjugated rabbit anti-TLR2 polyclonal antibody (bs-1019R-Alexa488, Bioss Inc., Wobum, MA, USA) or Alexa 488 conjugated rabbit anti-TLR4 antibody (bs-1021R-Alexa488, Bioss) diluted 50 times with Can Get Signal solution 1 (NKB-201, TOYOBO Co., Ltd., Osaka, Japan) overnight at 4°C. Both anti-TLR2 and anti-TLR4 antibodies cross-react with bovine receptors according to Bioss Inc. datasheet. Alexa 488 conjugate rabbit IgG (20304AF488, IMGENEX, San Diego, CA, USA) was used as isotype control. Following washing three times with PBS-T and the cells were rinsed in distilled water and then mounted with FLUOROSHIELD with DAPI (AR-6501-01, ImmunoBioScience Corp, Mukilteo, WA, USA). Immunofluorescence microscopy was performed with using a confocal laser microscope (LSM 700, Carl Zeiss, Oberkochen, Germany).

### Quantitative expression analysis of toll-like receptors by real-time polymerase chain reactions (PCR) in BIE cells

We performed two-step real-time quantitative PCR to characterize the expression of TLRs mRNAs in BIE cells. Total RNA from each sample was isolated from the BIE cells using TRIzol reagent (Invitrogen). All cDNAs were synthesized from 5 μg of total RNA using a Quantitect Reverse Transcription kit (Qiagen, Tokyo, Japan) according to the manufacturer’s recommendations. Real-time quantitative PCR was carried out using a 7300 Real-time PCR System (Applied Biosystems, Warrington, UK) using Platinum SYBR Green qPCR SuperMix Uracil-DNA Glycosylase (UDG) with Fast Start Universal SYBR Green Master (ROX) (Invitrogen). The primers for TLRs used in this study are described in Table 1. The PCR cycling conditions were 5 min at 50°C; followed by 5 min at 95°C; then 40 cycles of 15 sec at 95°C, 30 sec at 60°C and 30 sec at 72°C. The reaction mixture contained 5 μl of the sample cDNA and 15 μl of the master mix including the sense and antisense primers. Expression of β-actin was used to normalize cDNA levels for differences in total cDNA levels in the samples. TLRs mRNA levels in BIE cells were calibrated by the bovine β-actin level, and normalized by common logarithmic transformation in comparison to the TLR1 mRNA level in BIE cells (as 1.00).

**Table 1:** Primer sequences used in this study

**Primer**	**Sense primer**	**Antisense primer**	**Accession number**
β-actin	TGG ATT GGC GGC TCC AT	GCT GAT CCA CAT CTG CTG GAA	NM_173979
TLR1	CAT TCC TAG CAG CTA CCA CAA GCT	TGG GCC ATT CCA AAT AAG TTC T	NM_001046504
TLR2	GGG TGC TGT GTC ACC GTT TC	GCC ACG CCC ACA TCA TCT	NM_174197
TLR3	GGG CAC CTG GAG GTC CTT	TTC CTG GCC TGT GAG TTC TTG	NM_001008664
TLR4	AGC ACC TAT GAT GCC TTT GTC A	GTT CAT TCC GCA CCC AGT CT	NM_174198
TLR5	GTC CCC AAC ACC ACC AAG AG	GCG GTT GTG ACT GTC CTG ATA TAG	NM_001040501
TLR6	TTT ACC CTC AAC CAC GTG GAA	GGG CCA AAG GAA CTG AAA AAC	NM_001001159
TLR7	CAC CAA CCT TAC CCT CAC CAT T	GTC CAG CCG GTG AAA GGA	NM_001033761
TLR8	TGT GTT TAG AGG AAA GGG ATT GG	TCT GCA TGA GGT TGT CGA TGA	NM_001033937
TLR9	CAG TGG CCA GGG TAG TTT CTG	CCG GTT ATA GAA GTG ACG GTT GT	NM_183081
TLR10	TCT ACT GCA TCC CTA CCA GAT ATC C	GGG CCA TTC CAA GTA TGC TTT	NM_001076918
MCP-1	CAC CAG CAG CAA GTG TCC TAA A	CAC ATA ACT CCT TGC CCA GGA T	NM_174006
TNF-α	CGC ATT GCA GTC TCC TAC CA	GGG CTC TTG ATG GCA GAC A	NM_173966
TGF-β	CGT GGA GCT GTA CCA GAA ATA TAG C	CGA GCA GCC GGT TGC T	NM_001166068
IFN-α	GGT GGC AGC CAG TTA CAG AAG	TGC TGG GTC ACC TCA TGG A	Z46508
IFN-β	CGA TGG TTC TCC TGC TGT GTT	GAG CAA GCT GTA GCT CCT GGA A	EU276065
IFN-γ	GGA GGA CTT CAA AAA GCT GAT TCA	GGC TTT GCG CTG GAT CTG	NM_174086
LIF	CTG TCC CAG CAA CCT CAT GA	TGG CAC TGC TGT TGA GTT GTC	NM_173931
IL-1α	CAG TTG CCC ATC CAA AGT TGT T	TGC CAT GTG CAC CAA TTT TT	NM_174092
IL-1β	GAG CCT GTC ATC TTC GAA ACG	GCA CGG GTG CGT CAC A	NM_174093
IL-4	GCC ACA CGT GCT TGA ACA AA	TGC CAA GCT GTT GAG ATT CCT	NM_173921
IL-6	CCA CCC CAG GCA GAC TAC TTC	CCA TGC GCT TAA TGA GAG CTT	NM_173923
IL-7	CAA GCT TCA CCT ATC AAC AGT TTC A	CCC TTG CTG GTG CAG TTC A	NM_173924
IL-8	TGC TCT CTT GGC AGC TTT CC	TCT TGA CAG AAC TGC AGC TTC AC	NM_173925
IL-10	GGC GGT GGA GAA GGT GAA	GGC TTT GTA GAC ACC CCT CTC TT	NM_174088
IL-12	CAG CAA GCC CAG GAA GGA	TGA CAG CCC TCA GCA GGT TT	NM_174355
MKP-1	CGCAGCGCGCAAATCT	CGGGTAGGAAGCAGAAAAAGC	NM_001046452
IRAK-M	ACAGCGGAGCGGCTTTC	CTTGGTCTACATATTTTTCAATGTGA	NM_001190299
SIGIRR	GGCAGTGAAGTGGATGTGTCA	TCCGTGCGGGCACTGTA	NM_001082443
BCL3	CATGGAACACCCCCTGTCA	GGCGTATCTCCATCCTCATCA	NM_001205993
Tollip	CGGGCGTGGACTCTTTCTAC	GATGCGGTCGTCCATGGA	NM_001039961
ABIN-3	CGCAGAACGAATTGCTGAAA	CACTACGCTCCCTCTGGAAGTC	BC102932

### Anti-inflammatory assay in BIE cells

Lactobacilli were re-suspended in DMEM (10% FCS, 1% SP), enumerated in a microscope using a Petroff-Hausser counting chamber, and stored at −80°C until use. BIE cells were plated at 3×10^4^ cells/well of a 12-well ptype I collagen-coated plates (Iwaki, Tokyo, Japan), and cultured for three days. After changing medium, lactobacilli (5×10^7^ cells/ml) were added and 48 hours later, each well was washed vigorously with medium at least 3 times to eliminate all the stimulants. Expression of cytokines, chemokines and TLRs negative regulators were studied first without any inflammatory challenge by using real time PCR as described below. In addition, the effect of lactobacilli on BIE cells immune response was studied using heat-stable ETEC as inflammatory factor. BIE cells were treated with heat-stable ETEC (final concentration: 5×10^7^ cells/ml) for indicated time and the expression of cytokines, chemokines and TLRs negative regulators were studied by using real time PCR as described below. In addition, activation of p38, c-Jun N-terminal kinase (JNK) and extracellular signal-regulated kinase (ERK) mitogen-activated protein kinases and NF-кB pathways were studied by using western blotting as described below. In these experiments, the synthetic TLR2 agonist tripalmitoylated lipopeptide Pam3CysSerLys4 (Pam3CSK4) was also used. BIE cells were stimulated with Pam3CSK4 (final concentration: 200 ng/ml) for the indicated time same as the other stimuli.

### Quantitative expression analysis of cytokines, chemokines and TLRs negative regulators by PCR in BIE cells

Two-step real-time quantitative PCR was used to characterize the expression of cytokines, chemokines and TLRs negative regulators mRNAs in BIE cells. Total RNA from each sample was isolated from the BIE cells using TRIzol reagent (Invitrogen). All cDNAs were synthesized from 5 μg of total RNA using a Quantitect Reverse Transcription kit (Qiagen, Tokyo, Japan) according to the manufacturer’s recommendations. Real-time quantitative PCR was carried out using a 7300 Real-time PCR System (Applied Biosystems, Warrington, UK) using Platinum SYBR Green qPCR SuperMix UDG with ROX (Invitrogen). The primers for cytokines, chemokines and TLRs negative regulators used in this study are described in Table 1. The PCR cycling conditions were 5 min at 50°C; followed by 5 min at 95°C; then 40 cycles of 15 sec at 95°C, 30 sec at 60°C and 30 sec at 72°C. The reaction mixture contained 5 μl of the sample cDNA and 15 μl of the master mix including the sense and antisense primers. Expression of β-actin was used to normalize cDNA levels for differences in total cDNA levels in the samples. TLRs mRNA levels in BIE cells were calibrated by the bovine β-actin level, and normalized by common logarithmic transformation in comparison to the each control (as 1.00).

### Enzyme linked immunosorbent assay (ELISA) for the detection of cytokines

BIE cells were stimulated with *L*. *casei* OLL2768 or MEP221108 (5×10^7^ cells/ml) for 48 hr and then challenged with heat-stable ETEC PAMPs as described before. The concentration of IL-6 and MCP-1 secreted into the supernatant of BIE cell cultures was determined using two commercially available enzyme- linked immunosorbent assay (ELISA) kits (bovine IL-6 [ESS0029, Thermo Scientific, Rockford, IL, USA] and bovine CCL2/MCP-1 [E11-800, Bethyl Laboratories, Inc. Montgomery, TX, USA]), according to the manufacturers’ instructions.

### Western Blotting

BIE cells cultured in 1.8×10^5^ cells/60 mm dishes were stimulated with *Lactobacillus casei* OLL2768 or Pam3CSK4 with same time schedule and equivalent amount as mentioned above. BIE cells were then washed and stimulated with heat-stable ETEC PAMPs for indicated time. After stimulation, BIE cells were washed three times with PBS and resuspended in 200 μl of CelLytic M Cell Lysis Reagent (Sigma-Aldrich, St. Louis, MO, USA) including protease and phosphates inhibitors (complete Mini, PhosSTOP: Roche, Mannheim, Germany). Protein concentration was measured with BCA protein assay kit (Pierce, Rockford, IL, USA). Extracts (120 μl) were transferred into Eppendorf tubes and were added with 40 μl of Sample Buffer Solution (2ME+)(×4)(Wako), and boiled for 5 min at 95°C. Equal amounts of extracted proteins (2 μg) were loaded on 10% SDS-polyacrylamide gel electrophoresis (SDS-PAGE). Separated proteins were transferred electrophoretically to a PVDF membrane. The membrane was blocked with 2% BSA/TBS-T (w/v) for 2 hours at room temperature. Phosphorylation of p38, JNK and ERK mitogen-activated protein kinases and nuclear factor kappa B inhibitor protein (IkB) degradation were evaluated using Phospho-p38 MAPK (Thr180/Tyr182) antibody (p-p38, Cat. #9211); p38 MAPK antibody (p38, Cat. #9212); Phospho-SAPK/JNK (Thr183/Tyr185) antibody (p-JNK, Cat. #9251); SAPK/JNK antibody (JNK, Cat. #9252); Phospho-p44/42 MAP kinase (Thr202/Thy204) antibody (p-ERK, Cat. #9101); p44/42 MAP (Erk 1/2) antibody (ERK, Cat. #9102) and; I kappaB-alpha antibody (IkBa, Cat. #9242) from Cell Signaling Technology (Beverly, MA, USA) at 1000 times dilution of their original antibodies and with immunoreaction enhancer (Can Get Signal® Solution 1, TOYOBO Co. Ltd., Osaka, Japan) overnight at room temperature. After washing with TBS-T, the membrane was incubated alkaline phosphatase conjugated anti-rabbit IgG (Cat. #A3937, Sigma) at 2000 times dilution with immunoreaction enhancer (Can Get Signal® Solution 2, TOYOBO Co. Ltd., Osaka, Japan) for 1 hour at room temperature. After washing with TBS-T, signals were generated by overlaying the membrane with ECFTM substrate (GE Healthcare, Piscataway, NJ, USA) for 5 min at room temperature under dark conditions. The Attophos (Ex; 440nm, Em; 560nm) was detected by Molecular Imager Fx (Bio-Rad, Hercules, CA, USA). The densitometry of western blots was carried out by using Image J software (National Institutes of Health, Bethesda, MD, USA).

### Statistical analysis

Normalized relative expression of each control data (showed as the ratio to β-actin mRNA expression) was transferred to a normal distribution with a mean of 1.0. In order to normalize the control data, they were fitted by using the following function:

$$Z\left(x_{i}\right)=\left(x_{i}-x_{\left(\mathrm{control}\right)}-\sigma_{x}\right)/\sigma_{x}$$

Z(x_i_): all adjusted data; x_i_: i^th^ experimental data, x_(control)_: a mean of repeated control data; and σ_x_ was a standard deviation of repeated control or trial data. Similarly, normalized relative expression for heat-stable ETEC PAMPs and lactobacilli data was fitted to this function to show them as a fold value compared to the control data. Each of data number repeated in a same condition was from 8 to 10. Statistical analysis was performed by using SAS computer program, ver.6.0 and GLM procedure. The multiple comparisons among means of fold expression were carried out by Fisher’s least significance differential test, LSD method. Differences were significant at 5% level and were showed in graphs with superscripts letters (for differences between means) or asterisks (for differences between each treatment a control).

## Results

### Expression of TLRs in BIE cells

**Figure 1:**
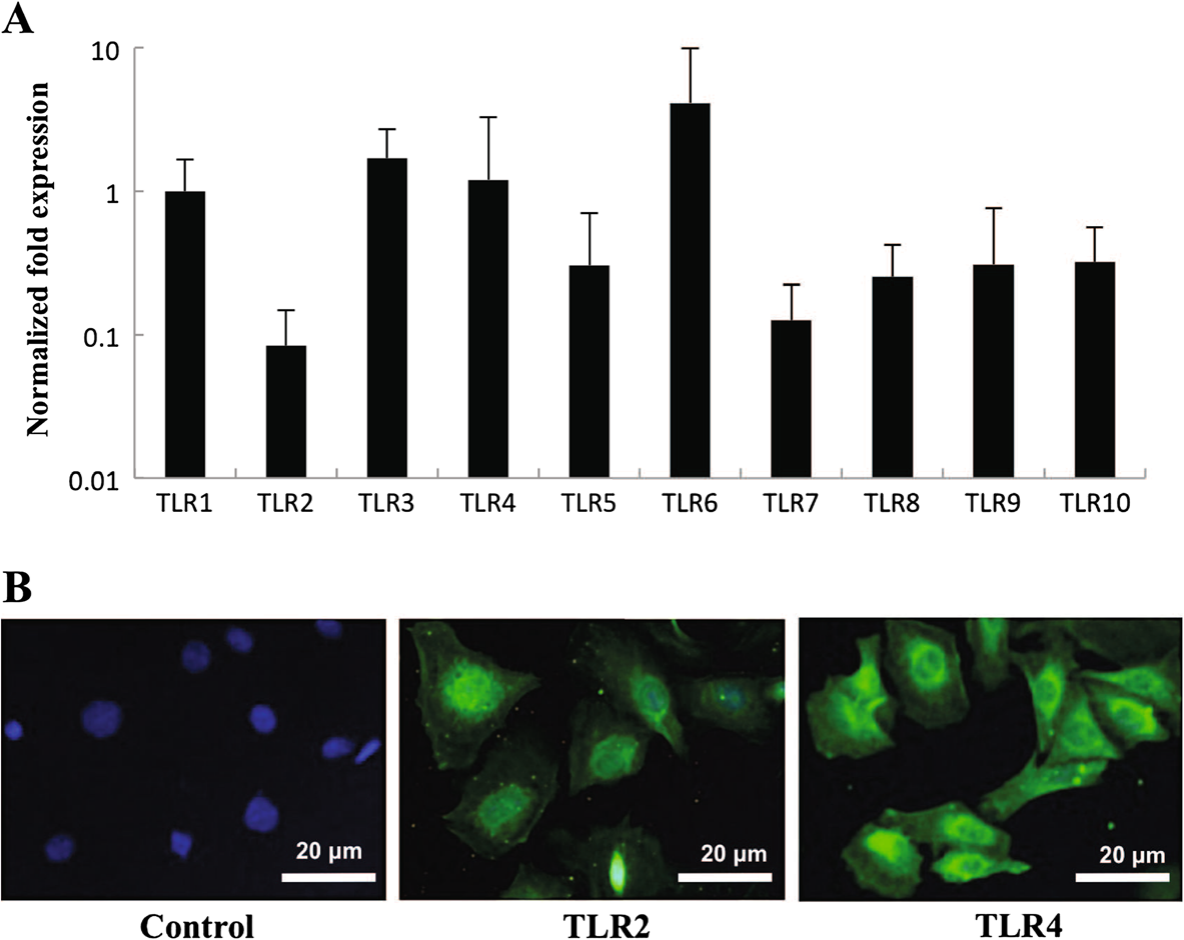
**Analysis of toll**-**like receptors (TLRs) expression in bovine intestinal epithelial (BIE) cells.** (**A**) TLR1-10 mRNA levels in BIE cells. The expression of TLR in BIE cells was calculated first as relative units compared to bovine β-actin level. After calculating the relative unit to β-actin, TLR1 was set as 1. Values represent means and error bars indicate the standard deviations. The results are means of six independent experiments. (**B**) Immunofluorescent localization of TLR2 and TLR4 in BIE cells. Green images indicate bovine TLR2 or TLR4 positive cells and nuclei in all panels were stained with DAPI (blue). Control experiments were performed by omitting the primary antibody. The results represent six independent experiments.

In order to study the mechanisms by which bovine IECs induce immune responses against intestinal pathogens, we have previously established a clonal bovine intestinal epithelial cell line (BIE cells). When BIE cells are cultured they assume monolayer cobblestone and epithelial-like morphology with close contact between the cells [17]. Moreover, scanning electron microscopy examination of BIE cell reveled that 3-days old cells have irregular and slender microvilli-like structures on their surface and that this structures increase in complexity as the cells grow [17]. In this work, we applied real-time quantitaive PCR to analyze the expression of TLRs mRNA in BIE cells. All TLRs genes were expressed in BIE cells (Figure 1A). Among TLR family, TLR1, 3, 4 and 6 were strongly expressed, followed by TLR5, 8, 9, 10, 2 and 7. We were particularly interested in expression of TLR2 and TLR4 as the main receptors detecting LAB and ETEC respectively. Therefore, to confirm these real-time PCR findings, we further examined the expression of TLR2 and 4 proteins in BIE cells using anti-TLRs antibodies that are able to cross-react with bovine TLRs (Figure 1B). Visualization of the immunofluorescense staining confirmed the protein expression of TLR2 and 4 in BIE cells (Figure 1B).

### Study of the inflammatory response in BIE cells stimulated with heat-stable ETEC PAMPs

**Figure 2:**
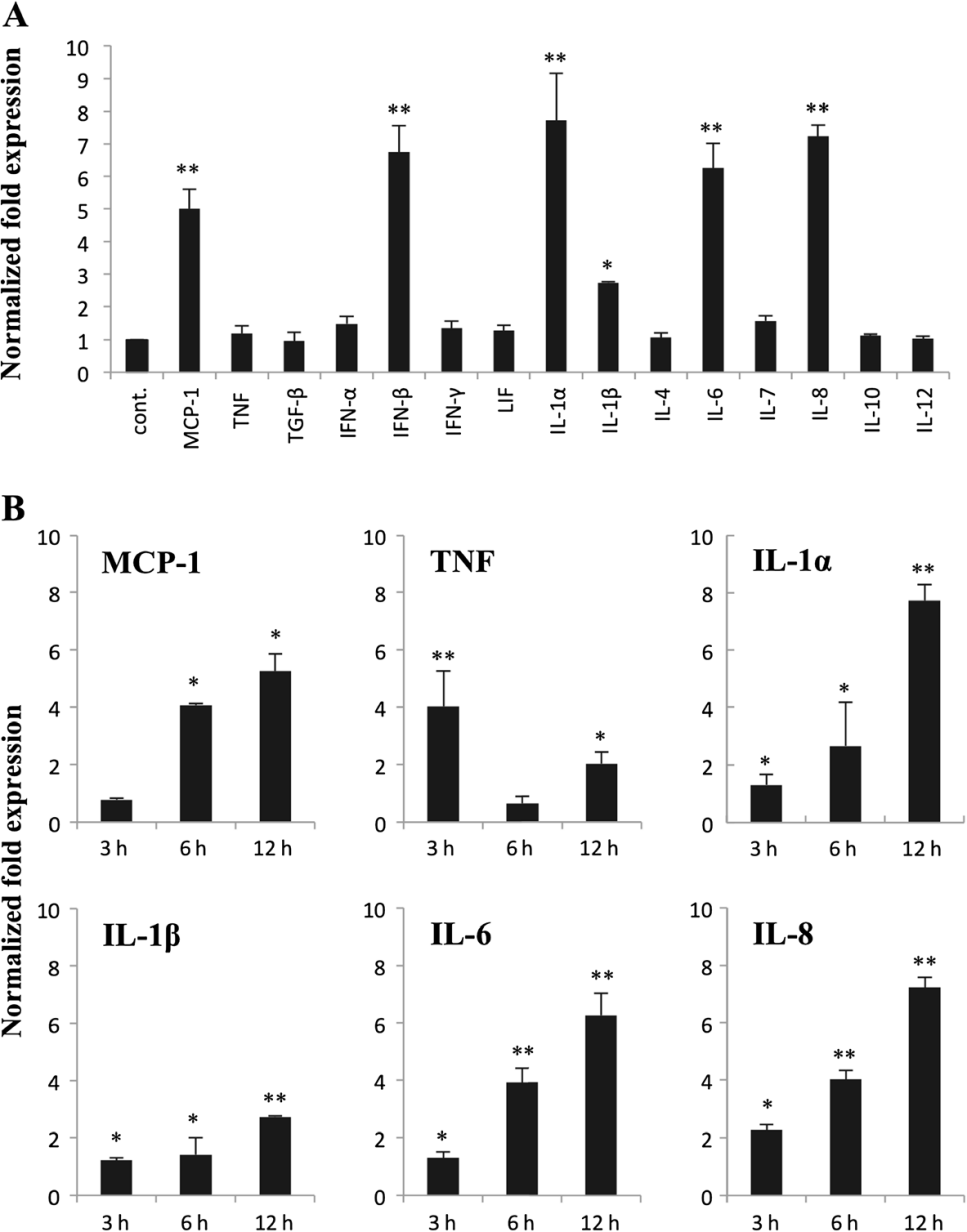
**Expression of cytokines in bovine intestinal epithelial (BIE) cells after stimulation with heat**-**stable Enterotoxigenic *Escherichia coli *(ETEC) pathogen**-**associated molecular patterns (PAMPs).** (**A**) BIE cells were challenged with heat-stable ETEC PAMPs and twelve hours later the expression of several cytokines was studied. The results represent four independent experiments. Significantly different from control *(P<0.05), **(P<0.01). (**B**) BIE cells were challenged with heat-stable ETEC PAMPs and the expression of MCP-1, TNF, IL-1-α, IL-β, IL-6 and IL-8 was studied at the indicated times post-stimulation. The results represent four independent experiments. Significantly different from time 0 *(P<0.05), **(P<0.01).

We next investigated the response of BIE cells to heat-stable ETEC PAMPs challenge. The ETEC 987P strain used in this study does not express flagellin and we have demonstrated that the main molecule responsible for the inflammatory response triggered by this bacterium is the LPS present on its surface [14,15]. BIE cells were cultured for 3 days and then challenged with heat-stable ETEC PAMPs. Twelve hours after stimulation we determined mRNA levels of several cytokines (Figure 2A). Stimulation of BIE cells with heat-stable ETEC PAMPs significantly increased the expression of pro-inflammatory cytokines MCP-1, IL-1α, IL-1β, IL-6 and IL-8 and the levels of IFN-β (Figure 2A). We also evaluated the mRNA levels of IL-1α, IL-1β, IL-6IL-8, TNF and MCP-1 at different times after stimulation with heat-stable ETEC PAMPs, with the aim of establishing the most appropriate time to study the inflammatory response. After the challenge with heat-stable ETEC PAMPs, levels of IL-1α, IL-1β, IL-6, IL-8, and MCP-1 increased progressively in BIE cells until the hour 12 post-stimulation (Figure 2B). On the contrary, mRNA levels of TNF in BIE cells stimulated with heat-stable ETEC PAMPs were increased earlier at hour 3 (Figure 2B). Considering these results, we selected the hour 12 post-stimulation for the following experiments.

### Selection of lactobacilli strains able to modulate inflammatory response in BIE cells

**Figure 3:**
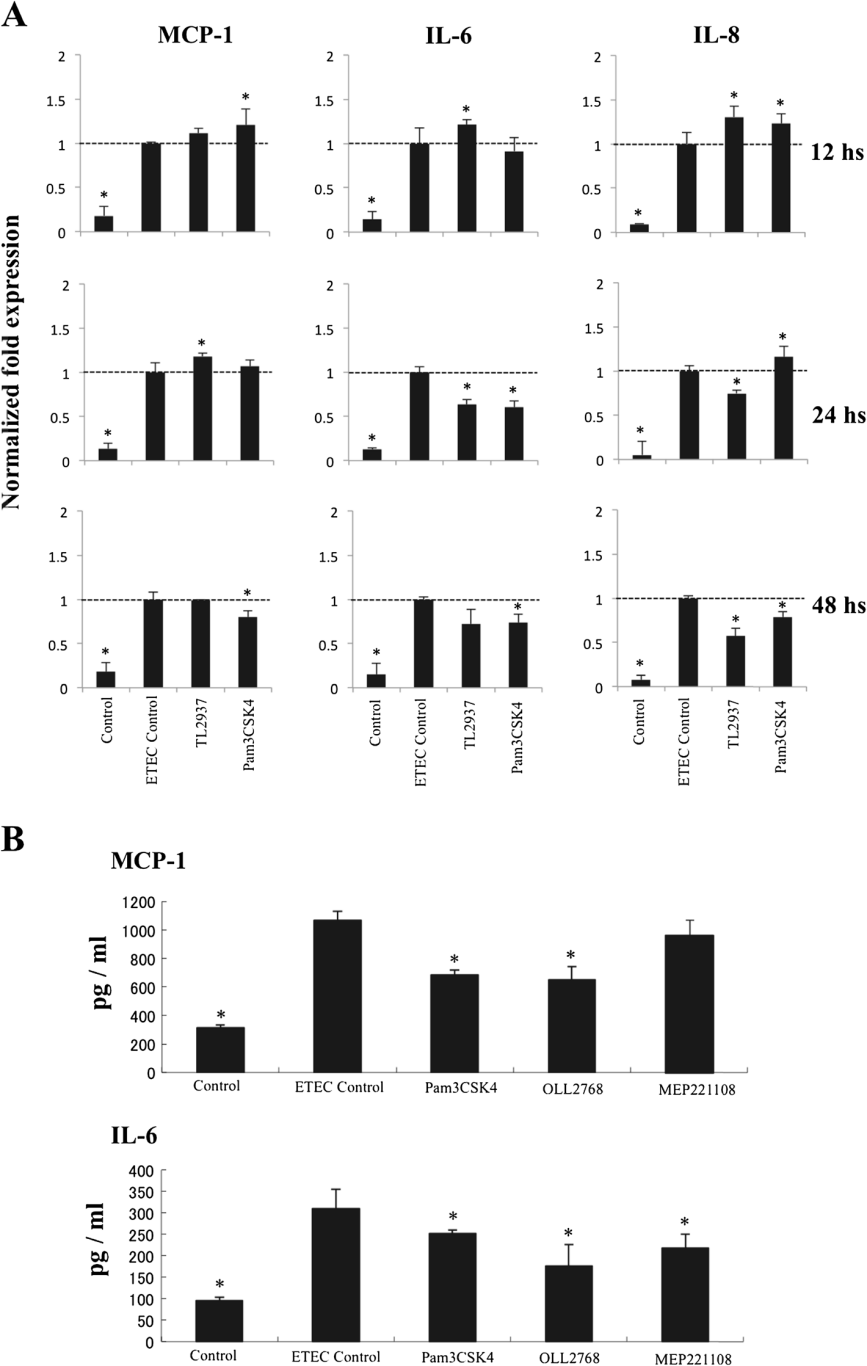
**Evaluation of the immunomodulatory activity of lactobacilli.** (**A**) Bovine intestinal epithelial (BIE) cells were pre-treated with immunobiotic *Lactobacillus jensenii* TL2937 or Pam3CSK4 for 12, 24 or 48 hours, stimulated with heat-stable ETEC PAMPs and then the expression of MCP-1, IL-6 and IL-8 was studied at hour twelve post-stimulation. Significantly different from ETEC Control *(P<0.05). (**B**) Levels of MCP-1 and IL-6 proteins. BIE cells were pre-treated with *Lactobacillus casei* OLL2768 or *L*. *casei* MEP221108 for 48 hours and the stimulated with heat-stable ETEC PAMPs and then levels of MCP-1 and IL-6 was studied at hour twelve post-stimulation. Significantly different from ETEC Control *(P<0.05).

Recently, we have demonstrated that stimulation with *Lactobacillus jensenii* TL2937 is able to down-regulate the levels of IL-6, IL-8 and MCP-1 produced by porcine IECs in response to heat-stable ETEC PAMPs or LPS challenges [14]. Moreover, we demonstrated that TLR2 is partially involved in this immunoregulatory effect of *L*. *jensenii* TL2937 in PIE cells [14]. Then, we next aimed to evaluate if this immunobiotic strain has a similar effect on BIE cells. For this reason, BIE cells were stimulated for 12, 24 or 48 hours with *L*. *jensenii* TL2937 or the synthetic TLR2 agonist Pam3CSK4 and then challenged with heat-stable ETEC PAMPs. Twelve hours after stimulation levels of MCP-1, IL-8 and IL-6 were evaluated (Figure 3A). Stimulation of BIE cells for 12 h with *L*. *jensenii* TL2937 or Pam3CSK4 significantly increased the production of IL-8 in response to heat-stable ETEC PAMPs challenge in hour 12 post-stimulation. On the contrary, levels of IL-8 were significantly lower in cells treated for 48 h with *L*. *jensenii* TL2937 or Pam3CSK4. MCP-1 levels were significantly higher than controls in BIE cells treated for 12 h with Pam3CSK4 or 24 h with *L*. *jensenii* TL2937 (Figure 3A). BIE cells pre-stimulated with *L*. *jensenii* TL2937 or Pam3CSK4 during 24 h showed significantly reduced levels of IL-6 (Figure 3A).

These results indicate that it is possible to modulate the inflammatory response in BIE cells by using LAB. Then, we next aimed to evaluate the potential anti-inflammatory effect of 20 lactobacilli strains in BIE cells with the aim of finding the strain with the highest immunomodulatory capacity in the bovine system. First, we evaluated the effect of lactobacilli on BIE cells without any inflammatory challenge (Additional file 1: Figure S1A). BIE cells were treated with the different lactobacilli strains for 48 h and the levels of mRNA IL-6, IL-8 and MCP-1 were determined. Only the strain MEP221102 slightly increased levels of MCP-1, and MEP221108 and MEP221114 also slightly increased levels of IL-6 in BIE cells (Additional file 1: Figure S1A). On the contrary, several strains were able to significantly down-regulate the levels of IL-8 in BIE cells (Additional file 1: Figure S1A). Next, we evaluated the effect of lactobacilli on BIE cells using heat-stable ETEC PAMPs as inflammatory factor (Additional file 1: Figure S1B). For this purpose, BIE cells were stimulated with the different LAB strains for 48 h, challenged with heat-stable ETEC PAMPs and the levels of the three pro-inflammatory cytokines were studied at hour 12 post-stimulation. MCP-1, IL-6 and IL-8 levels in BIE cells stimulated with OLL2768, MEP221101, MEP221105 and MEP221111 strains were significantly lower than those observed in the control. On the contrary, the other strains tested reduced one of the cytokines studied or had no effect (Additional file 1: Figure S1B). Considering that *L*. *casei* OLL2768 and *L*. *casei* MEP221111 showed the highest capacity to down-regulate IL-8 and also were able to reduce IL-6 and MCP-1 after heat-stable ETEC PAMPs challenge, one of these strains (*L*. *casei* OLL2768) was selected for the following experiments. To further confirm the immunoregulatory effect of *L*. *casei* OLL2768 and to obtain transcriptional data supported by protein detection of selected cytokines, we conducted ELISAs to evaluate the levels of IL-6 and MCP-1 proteins (Figure 3B). BIE cells were stimulated with *L*. *casei* OLL2768 or *L*. *casei* MEP221108 (negative control) and 48 h after were challenged with heat-stable ETEC PAMPs. Challenge significantly increased levels of both IL-6 and MCP-1 proteins. Pretreatment of BIE cells with *L*. *casei* OLL2768 significantly reduced levels of MCP-1, however *L*. *casei* MEP221108 was not able to modify MCP-1 values (Figure 3B). Both *L*. *casei* OLL2768 and MEP221108 were able to reduce levels of IL-6 after the challenge with heat-stable ETEC PAMPs, however the effect of *L*. *casei* OLL2768 was significantly higher than those observed for MEP221108. In addition, we evaluated if the TLR2 agonist Pam3CSK4 was able to modulate IL-6 and MCP-1 synthesis. BIE cells pretreated Pam3CSK4 showed reduced levels of both cytokines after heat-stable ETEC PAMPs challenge (Figure 3B).

### Effect of L. casei OLL2768 on MAPK and NF-κB pathways in BIE cells

**Figure 4:**
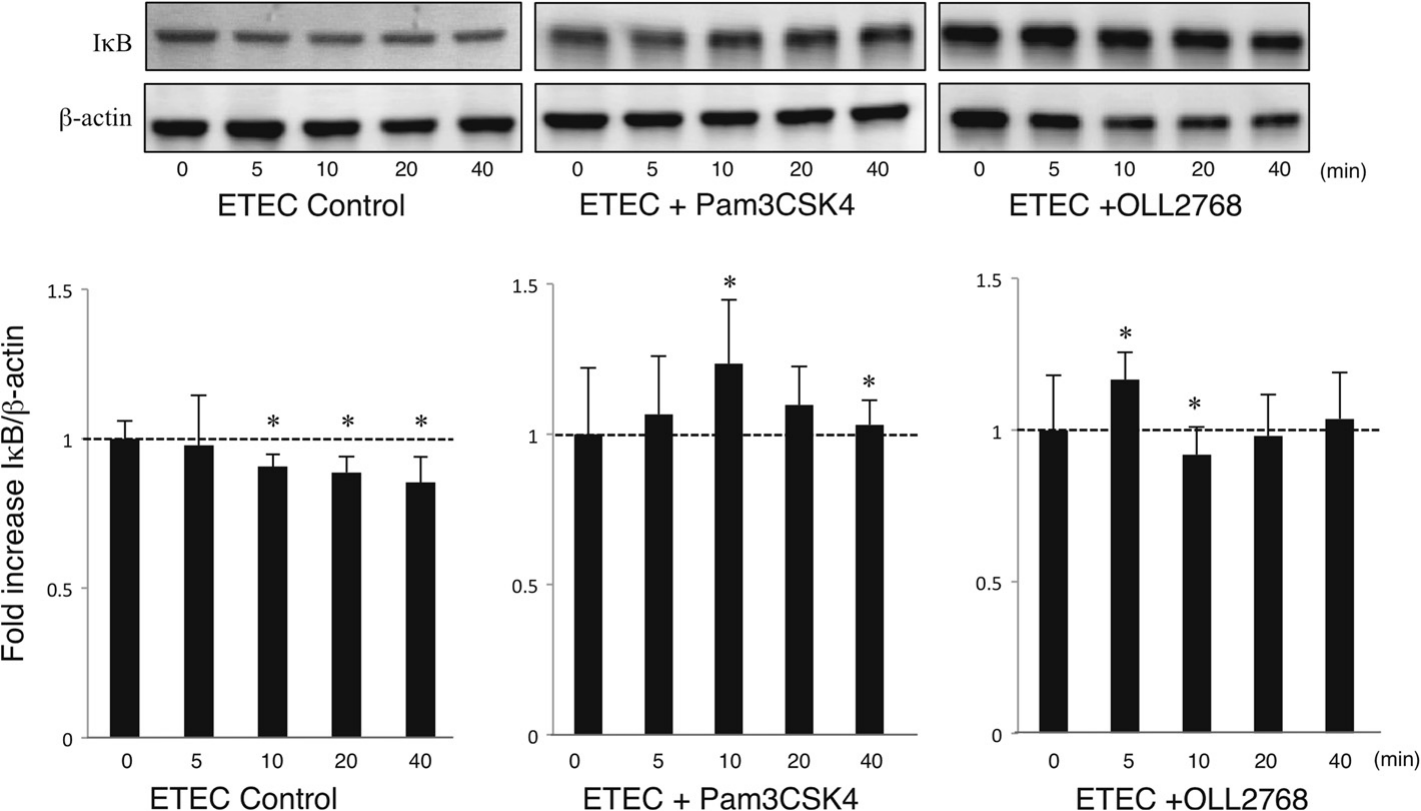
**Western blot analysis of IκB degradation on bovine intestinal epithelial (BIE) cells after challenge with heat**-**stable Enterotoxigenic *Escherichia coli *(ETEC) pathogen**-**associated molecular patterns (PAMPs).** BIE cells were pre-treated with *Lactobacillus casei* OLL2768 or Pam3CSK4 for 48 hours and then stimulated with heat-stable ETEC PAMPs or LPS. Levels of the counter-regulatory factor IκBα were studied at the indicated times post-stimulation. Significantly different from time 0 *(P<0.05).

**Figure 5:**
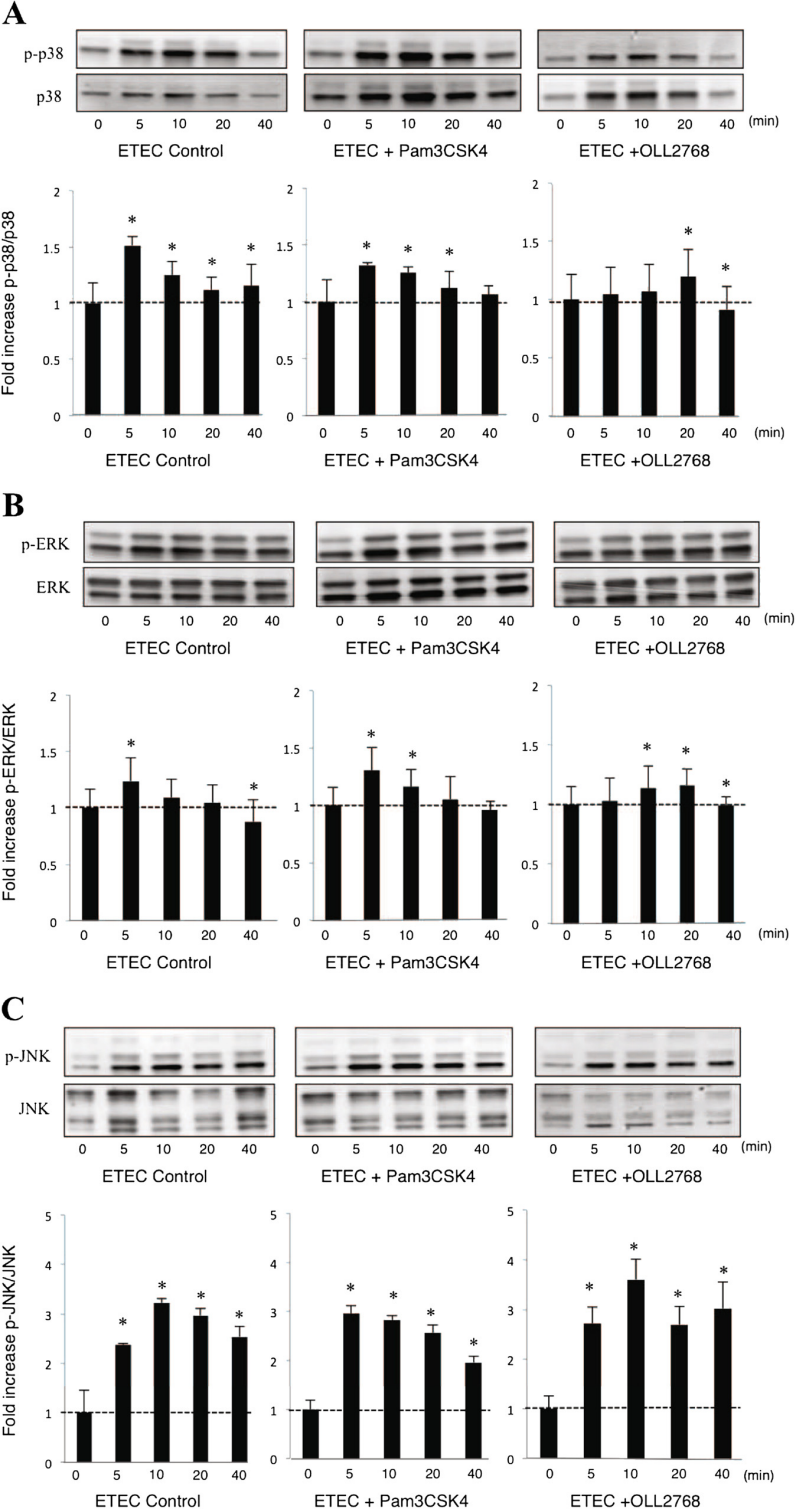
**Western blot analysis of p38**, **JNK and ERK mitogen**-**activated protein kinases activation on bovine intestinal epithelial (BIE) cells after challenge heat**-**stable Enterotoxigenic *Escherichia coli *(ETEC) pathogen**-**associated molecular patterns (PAMPs).** BIE cells were pre-treated with *Lactobacillus casei* OLL2768 or Pam3CSK4 for 48 hours and then stimulated with heat-stable ETEC PAMPs or LPS. Phosphorylation of p38, JNK and ERK was studied at the indicated times post-stimulation. Significantly different from time 0 *(P<0.05).

We next evaluated whether *L*. *casei* OLL2768 was able to attenuate heat-stable ETEC PAMPs-mediated pro-inflammatory responses by modulating the NF-κB pathway. Challenge of BIE cells with heat-stable ETEC PAMPs significantly reduced the levels of the counter-regulatory factor IκBα (Figure 4). BIE cells previously stimulated with *L*. *casei* OLL2768 or Pam3CSK4 did not show a significant degradation of IκBα indicating an inhibitory effect in NF-κB pathway (Figure 4). We also examined the relationship between MAPK activation and regulation of pro-inflammatory cytokines in BIE cells by *L*. *casei* OLL2768 (Figure 5). BIE cells were stimulated with OLL2768 strain, Pam3CSK4 or control medium and the activation profiles of p38, ERK and JNK were compared. As shown in Figure 5A and B, heat-stable ETEC PAMPs induced the phosphorylation of p38 and ERK, which reached a maximum between 5 and 10 minutes. The time course of ERK phosphorylation induced by heat-stable ETEC PAMPs in BIE cells treated with Pam3CSK4 showed a similar tendency to that observed in the control. On the contrary, reduced phosphorylation of p38 was observed in Pam3CSK4- and *L*. *casei* OLL2768-treated BIE cells (Figure 5A, B). In addition, in *L*. *casei* OLL2768- treated BIE cells a delayed increase of p-ERK was observed when compared to control. In *L*. *casei* OLL2768-treated cells the levels of p-ERK were significantly increased 10 min after heat-stable ETEC PAMPs challenge (Figure 5C). The time course of JNK phosphorylation induced by heat-stable ETEC PAMPs in BIE cells treated with Pam3CSK4 showed a similar tendency to that observed in the control (Figure 5C). In *L*. *casei* OLL2768- treated BIE cells, phosphorylation of JNK significantly increased at minutes 5 and 10 after heat-stable ETEC PAMPs challenge. In addition, the levels of p-JNK decreased at minutes 20 and 40 in *L*. *casei* OLL2768-treated BIE cells, showing a difference with the control cells (Figure 5C).

### Effect of *L. casei* OLL2768 on negative regulators of the TLRs signaling pathway in BIE cells

**Figure 6:**
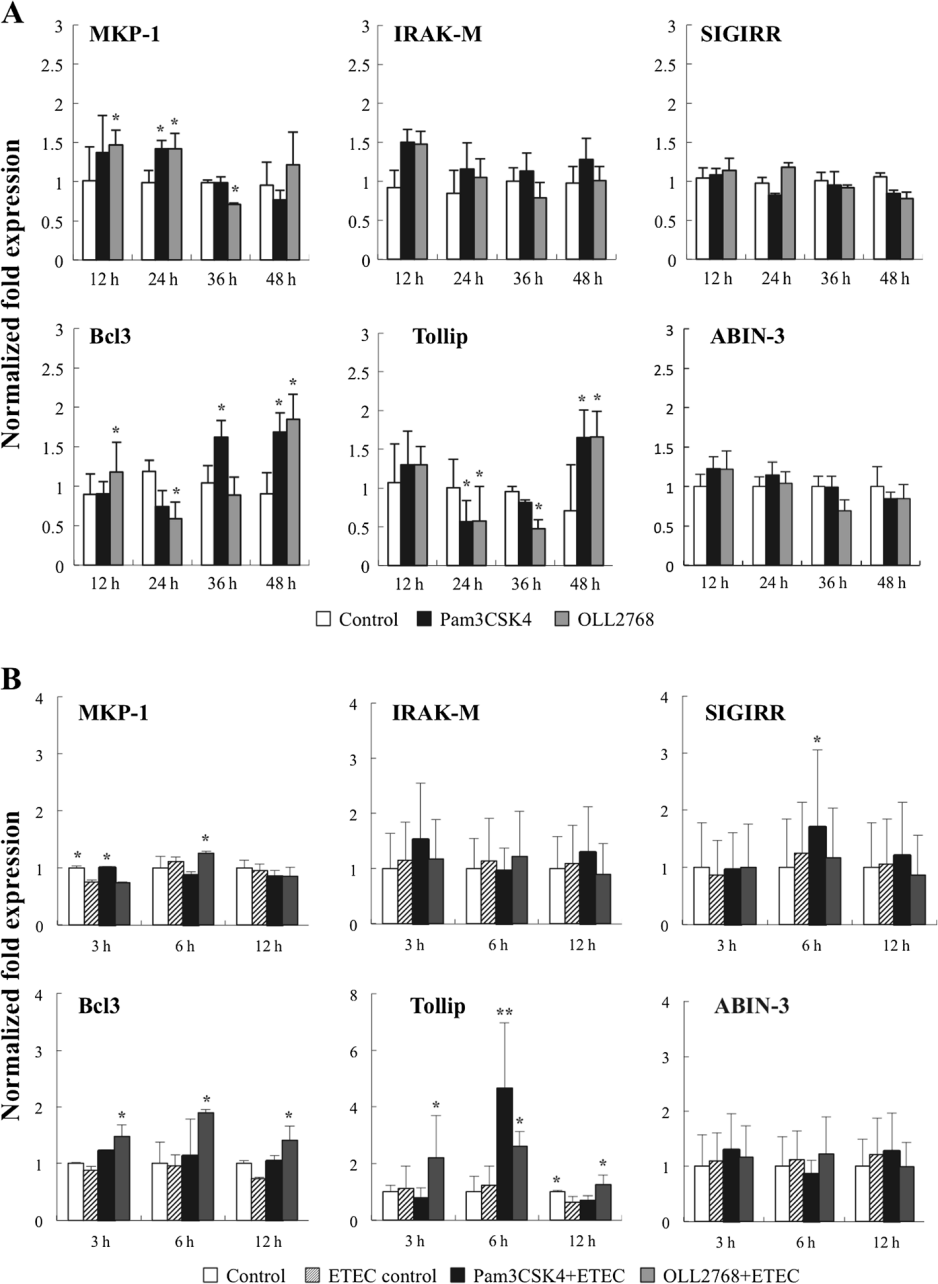
**Expression of toll**-**like receptor negative regulators in bovine intestinal epithelial (BIE) cells.** (**A**) BIE cells were stimulated with *Lactobacillus casei* OLL2768 or Pam3CSK4 for 12, 24, 36 or 48 hours and the expression of MKP-1, IRAK-M, SIGIRR, Bcl-3, Tollip and ABIN-3 negative regulators was studied. The results represent four independent experiments. Significantly different from control at the same time point *(P<0.05). (**B**) BIE cells were pre-treated with *Lactobacillus casei* OLL2768 or Pam3CSK4 for 48 hours and then stimulated with heat-stable Enterotoxigenic *Escherichia coli* (ETEC) pathogen-associated molecular patterns (PAMPs). The expression of MKP-1, IRAK-M, SIGIRR, Bcl-3, Tollip and ABIN-3 negative regulators was studied at the indicated times post-heat-stable ETEC PAMPs challenge. The results represent four independent experiments. Significantly different from ETEC control at the same time point *(P<0.05), **(P<0.01).

We studied the negative regulators that are known to mediate the TLR signaling pathway. First, we aimed to evaluate the changes in TLRs negative regulators without any pro-inflammatory challenge. For this reason, BIE cells were stimulated for 12, 24, 36 or 48 hours with *L*. *casei* OLL2768 or Pam3CSK4 and the expression of single immunoglobulin IL-1-related receptor (SIGIRR), Toll interacting protein (Tollip), A20-binding inhibitor of nuclear factor kappa B activation 3 (ABIN-3), B-cell lymphoma 3-encoded protein (Bcl-3), mitogen-activated protein kinase 1 (MKP-1) and interleukin-1 receptor-associated kinase M (IRAK-M) was determined by real-time PCR. None of the treatments were able to significantly induce changes in the expression of SIGIRR, ABIN-3 or IRAK-M (Figure 6A). We observed a slightly increase of MKP-1 after 24 hours of stimulation with both *L*. *casei* OLL2768 or Pam3CSK4, however this increase was not maintained after 36 hours. In addition, both treatments were capable of up-regulate the expression of Tollip after 48 h post-stimulation (Figure 6A). The expression of Bcl-3 was significantly up-regulated after 36 h post-stimulation with Pam3CSK4 or 48 h with Pam3CSK4 and *L*. *casei* OLL2768 (Figure 6A). We next evaluated the changes in the expression of TLR negative regulators after the challenge with heat-stable ETEC PAMPs. Again, BIE cells were treated with *L*. *casei* OLL2768 or Pam3CSK4 for 48 hours and stimulated with heat-stable ETEC PAMPs. No changes were observed in the expression of IRAK-M and ABIN-3 with either treatment (Figure 6B). MKP-1 was significantly up-regulated in OLL2768-treated BIE cells only in hour 6 post-challenge. In addition, the stimulation of BIE cells with Pam3CSK4 increased expression levels of SIGIRR and Tollip at hour 6 post-stimulation with heat-stable ETEC PAMPs. On the other hand, BIE cells treated with *L*. *casei* OLL2768 showed significantly higher levels of Bcl-3 and Tollip during all the studied period when compared to untreated control BIE cells (Figure 6B).

## Discussion

Although once considered simply a physical barrier, it is becoming increasingly evident that the epithelium plays as a crucial regulator of intestinal immune homeostasis. In response to invasive bacteria, IECs may produce a variety of cytokines and chemokines that play a crucial role in both the innate and adaptive immune responses in the gut [20]. In this paper, in order to understand the functional role of the bovine intestinal epithelium in mucosal host defense as part of the immune system, we studied in BIE cells the expression of TLRs and characterized heat-stable ETEC PAMPs-induced signal transduction pathways and cytokine induction. It is known that IECs are able to respond to pathogenic microorganisms because their expression of pattern recognition receptors (PRRs) such as TLRs. Therefore, the first aim of our research was to investigate the expression of TLRs in BIE cells. We found that TLR4 was one of the most highly expressed TLRs in BIE cells. TLR4 is conserved among different species and its expression appears to be a characteristic feature of IECs [21], therefore, the presence of TLR4 in BIE cells resembles IECs of other species.

The inflammatory response triggered by the activation of TLR4 in IECs play a critical role in host defense against Gram(−) pathogens. In this study, we showed that heat-stable ETEC PAMPs from strain 987P significantly enhanced the production of IL-6, IL-8, IL-1α and MCP-1 in BIE cells by activating both NF-κB and MAPK pathways. These findings correlate with our previous observations since we demonstrated that the heat-killed ETEC 987P strain, which does not express flagellin, triggers a TLR4-mediated inflammatory response in porcine intestinal epithelial cells through its LPS [21]. Moreover, the findings of the present work correlate with studies of the immune response against ETEC in IECs of different hosts species. It was shown that both NF-κB and MAPK pathways are important mediators of ETEC and LPS activation in human (HT29 and T84), mouse (CMT93) and porcine (PIE) IECs [14,22].

The cytokines produced by BIE cells may have an important protective role during ETEC infection. The enhanced secretion of IL-8 stimulates the strong infiltration of neutrophils in the lamina propria that is observed upon ETEC infection. Following IL-8 induced recruitment of neutrophils IL-6 can induce degranulation of these cells, thereby enhancing the inflammatory response [23]. On the other hand, IECs are able to produce MCP-1 in response to ETEC challenge. This chemokine has potent monocytes-activating and attracting propierties and plays a major role during intestinal inflammation [24]. Therefore, our findings indicate that BIE cells are useful cell line for studying inflammatory responses via TLR4 in vitro. Moreover, taking into consideration that inflammatory responses induced by intestinal pathogens can lead to dysregulation of IECs signaling, disruption of membrane barrier integrity, enhancement of pathogen translocation and disease [5], BIE cells could be also used to evaluate therapies designed for preventing inflammatory damage caused by heat-stable ETEC PAMPs during ETEC infection.

Several reports have demonstrated that immunobiotic LAB are able to improve resistance against pathogens and to protect against inflammatory damage caused by the infectious process [25-27]. Therefore we next aimed to evaluate if an immunobiotic lactobacillus strain could regulate the inflammatory response induced by heat-stable ETEC PAMPs in BIE cells. Our laboratory has recently found that *L*. *jensenii* TL2937 has a high capacity to down-regulate IL-6 and IL-8 production by PIE cells in response to heat-stable ETEC PAMPs or LPS challenges [14]. For these reasons, we first focused on *L*. *jensenii* TL2937 to evaluate its anti-inflammatory effect in BIE cells. *L*. *jensenii* TL2937 is able to decrease IL-6 (20% lower than control) and IL-8 (25% lower than control) expressions in heat-stable ETEC PAMPs-challenged BIE cells. However, this effect was lower when compared with the immunomodulatory activity of this strain in porcine IECs [14]. In heat-stable ETEC PAMPs-challenged porcine IECs previously treated with *L*. *jensenii* TL2937 the expression of IL-6 and IL-8 were 35% and 30% lower than control respectively [14]. Although the effect of *L*. *jensenii* TL2937 in BIE cells was lower than the previously described in porcine IECs, the present study indicate that LAB strains could be beneficial for attenuating inflammatory damage caused by heat-stable ETEC PAMPs in BIE cells. Thus, we next aimed to select the most effective strains of lactobacilli able to modulate heat-stable ETEC PAMPs-mediated inflammatory response in BIE cells. Several strains were evaluated in our system and we found that some lactobacilli were able to down-regulate the expression of inflammatory cytokines. Among these strains, *L*. *casei* OLL2768 showed the most pronounced effect. Of interest, we showed that the immunoregulatory effect of *L*. *casei* OLL2768 in BIE cells was more pronounced than that observed for *L*. *jensenii* TL2937, while the effect of OLL2768 strain was lower in porcine IECs [14]. Then, our findings indicate that is appropriate to evaluate different strains carefully according to the specific host, because the effect of the same LAB strain may differ according to the host that consumes it. In this sense, our in vitro bovine system can be of great value to find immunobiotic LAB strains suitable on the bovine host.

In BIE cells, *L*. *casei* OLL2768 attenuated heat-stable ETEC PAMPs-induced pro-inflammatory response and we confirmed that these effects were related to the capacity of OLL2768 strain to inhibit NF-κB and p38 signaling pathways in heat-stable ETEC PAMPs-challenged BIE cells. These results are reminiscent of other studies showing that probiotics are able to suppress TNF- or *S*. *typhimurium*- induced IL-8 gene expression and secretion by IECs in a NF-κB-dependent manner [28,29]. Moreover, our experiments extended these findings by showing that LAB are able to inhibit p38 signaling pathway in heat-stable ETEC PAMPs-challenged bovine IECs.

The JNK and p38 MAPK pathways share several upstream regulators, and accordingly there are multiple stimuli that simultaneously activate both pathways. Then we expected that *L*. *casei* OLL2768 had the same effect on JNK as they had in p38 pathway. However, we found an opposite behavior in JNK pathway. While in *L*. *casei* OLL2768-treated BIE cells the phosphorylation of p38 was reduced after challenge with heat-stable ETEC PAMPs, increased levels of p-JNK were detected. It was shown that these two stress-activated signaling pathways induce opposite effects and there is evidence indicating that the p38 MAPK pathway can negatively regulate JNK activity in several contexts [30,31]. In fact, the first evidence for this crosstalk was the observation that chemical inhibition of p38α and p38β strongly increased the activation of JNK, which was induced by IL-1 and sorbitol in epithelial cells and by LPS in macrophages [31]. Moreover, the kinetic analysis of our results showed an up-regulation of p-p38 between 5 and 10 minutes after heat-stable ETEC PAMPs challenge that was followed by a down-regulation of p-JNK between 10 and 20 minutes. Therefore, we can speculate that *L*. *casei* OLL2768 has a direct influence in p38 pathway while its effect in JNK is the result of the inhibition of p38 phosphorylation. Further research is needed to clarify completely the influence of *L*. *casei* OLL2768 in MAPK pathways in heat-stable ETEC PAMPs-challenged BIE cells.

Regulatory proteins can modulate the duration and intensity of TLRs signals [32]. Consequently, to dissect the mechanism(s) that underlie the anti-inflammatory effect of *L*. *casei* OLL2768, we evaluated the effect of this strain on the expression of the TLRs negative regulators in BIE cells. We observed that *L*. *casei* OLL2768 can negatively regulate TLR4 signaling in BIE cells by up-regulating Tollip and Bcl-3 proteins. Bcl-3 functions as an inhibitor of NF-κB activity by stabilizing repressive NF-κB homodimers in a DNA-bound state and preventing the binding of transcriptionally active dimers. In fact, stabilization of repressive complexes through the induction of Bcl-3 expression has been proposed to function in the processes of LPS tolerance [33]. On the other hand, it was demonstrated that overexpression of Tollip impairs TLR4-triggered NF-кB and MAPK signaling pathways and that inhibition of TLR signaling by Tollip is mediated through its ability to suppress the activity of IL-1 receptor-associated kinase (IRAK) [34,35]. Moreover, it was showed that prior exposure of IECs to a TLR ligand, such as LPS, induces a hyporesponsive state to a second challenge with the same or another TLR ligand by selectively limiting pro-inflammatory responses through up-regulation of Tollip and subsequent suppression of IRAK [35]. Therefore, the induction of Bcl-3 and Tollip by *L*. *casei* OLL2768 in BIE cells is important in establishing NF-κB- and MAPK-mediated tolerance against heat-stable ETEC PAMPs.

**Figure 7:**
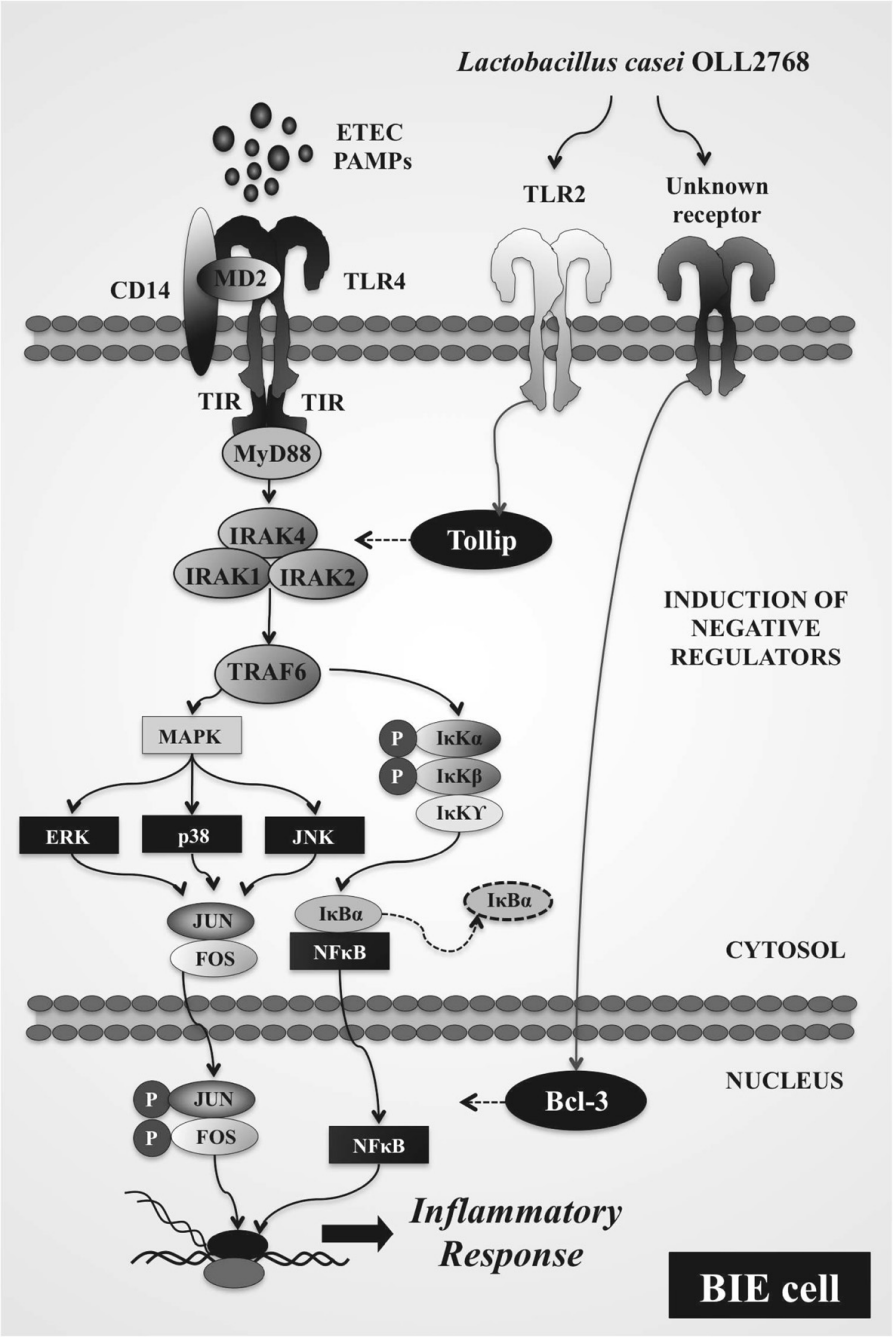
**Proposed mechanism for the anti**-**inflammatory effect of *Lactobacillus casei *OLL2768 in bovine intestinal epithelial (BIE) cells after challenge heat**-**stable Enterotoxigenic *Escherichia coli *(ETEC) pathogen**-**associated molecular patterns (PAMPs).**

At present, we cannot provide the conclusive mechanism for the anti-inflammatory action of *L*. *casei* OLL2768 on BIE cells. However, we can hypothesize that when *L*. *casei* OLL2768 encounters BIE cells it interacts with one or more PRRs and induces the up-regulation of Bcl-3 and Tollip negative regulators (Figure 7). Then, BIE cells pretreated with this immunobiotic strain produce lower concentrations of inflammatory mediators in response to heat-stable ETEC PAMPs challenge that could help to limit the inflammatory damage. One of the possible PRR involved in the anti-inflammatory effect of *L*. *casei* OLL2768 could be TLR2 since our comparative studies with Pam3CSK4 demonstrated that treatment of BIE cells with the TLR2 agonist up-regulate the expression of Tollip and reduce activation of NF-κB and p38 MAPK pathways. Moreover, it was found that LPS cross-tolerance may be induced by pre-exposure to lipoteichoic acid which leads to up-regulation of the common checkpoint Tollip via TLR2 [36]. However, further research is needed to resolve which PRR is activated by *L*. *casei* OLL2768 for the induction of negative regulators.

## Conclusion

We firstly reported in this study that BIE cells are useful for studying in vitro inflammatory responses in the bovine gut epithelium triggered by activation of TLR4. We also demonstrated that BIE cells can be used for the selection of immunomodulatory LAB and for studying the mechanisms involved in the protective activity of immunobiotics against pathogen-induced inflammatory damage, providing useful information that may be used for the development of new immunologically functional feeds through the screening and precise selection of lactobacilli strains that are able to beneficially modulate the immune system in the bovine host. In addition, we showed that *L*. *casei* OLL2768 functionally modulate the bovine intestinal epithelium by attenuating heat-stable ETEC PAMPs-induced NF-κB and MAPK activation and pro-inflammatory cytokines expression. Therefore *L*. *casei* OLL2768 is a good candidate for in vivo studying the protective effect of LAB against intestinal inflammatory damage induced by ETEC infection or heat-stable ETEC PAMPs challenge in the bovine host.

## Abbreviations

ABIN-3: A20-binding inhibitor of nuclear factor kappa B activation 3; Bcl-3: B-cell lymphoma 3-encoded protein; BIE cells: Bovine intestinal epithelial cell; DMEM: Dulbecco’s Modified Eagle media; ETEC: Enterotoxigenic *Escherichia coli*; ERK: Extracellular signal-regulated kinase; GLM: General Linear Models; HEKpTLR2: Porcine toll-like receptor 2-expressing transfectant; IRAK-M: Interleukin-1 receptor-associated kinase M; IECs: Intestinal epithelial cells; IL: Interleukin; IκB: Nuclear factor kappa B inhibitor protein; JNK: c-Jun N-terminal kinase; LAB: Lactic acid bacteria; LPS: Lipopolysaccharide; MAPK: Mitogen-activated protein kinases; MKP-1: Mitogen-activated protein kinase 1; MRS: Man-Rogosa-Sharpe; NF-κB: Nuclear factor kappa B; Pam3CSK4: Synthetic tripalmitoylated lipopeptide Pam3CysSerLys4; PAMP: Pathogen-associated molecular patterns; PBS: Phosphate-buffered saline; PIE: Porcine intestinal epithelial; PRR: Pattern recognition receptor; REG: Regression; ROX: Fast Start Universal SYBR Green Master; p38: p38 mitogen-activated protein kinase; SIGIRR: Single immunoglobulin IL-1-related receptor; TGF-β: Transforming growth factor β; TNF: Tumor necrosis factor; TLR: Toll-like receptor; Tollip: Toll interacting protein; UDG: Uracil-DNA Glycosylase

## Competing interests

The authors declare that they have no competing interests.

## Authors’ contributions

NT, YT, JV and HK conceived the study; NT, YT, JV, SI, HI, TS and HK designed the study; NT, YT, JV, KM, TT and EC did the laboratory work. NT, YT, JV, MT, TS, HA, YS, YK, HK analysed the data. NT, YT, JV and HK wrote the manuscript; all authors read and approved the manuscript.

## Authors’ information

Julio Villena: JSPS Postdoctoral Fellowship for Foreign Researchers.

## Supplementary Material

Additional file 1: Figure S1 Selection of immunomodulatory lactobacilli. (A) BIE cells were pre-treated with different lactobacilli strains for 48 hours and the expression of MCP-1, IL-6 and IL-8 was studied. Values represent means and error bars indicate the standard deviations. The results represent five independent experiments. Significantly different from control *(P<0.05). (B) BIE cells were pre-treated with different lactobacilli strains for 48 hours and the stimulated with heat-stable ETEC PAMPs and then the expression of MCP-1, IL-6 and IL-8 was studied at hour twelve post-stimulation. Values represent means and error bars indicate the standard deviations. The results represent five independent experiments. Significantly different from ETEC control *(P<0.05). Click here for file
