# Supplementary figures and images for: Advanced application of bovine intestinal epithelial cell line for evaluating regulatory effect of lactobacilli against heat-killed enterotoxigenic Escherichia coli-mediated inflammation
Source: BMC Microbiol. 2013 Mar 7;13:54. doi: 10.1186/1471-2180-13-54 (PMC3605377; doi:10.1186/1471-2180-13-54)

**A**

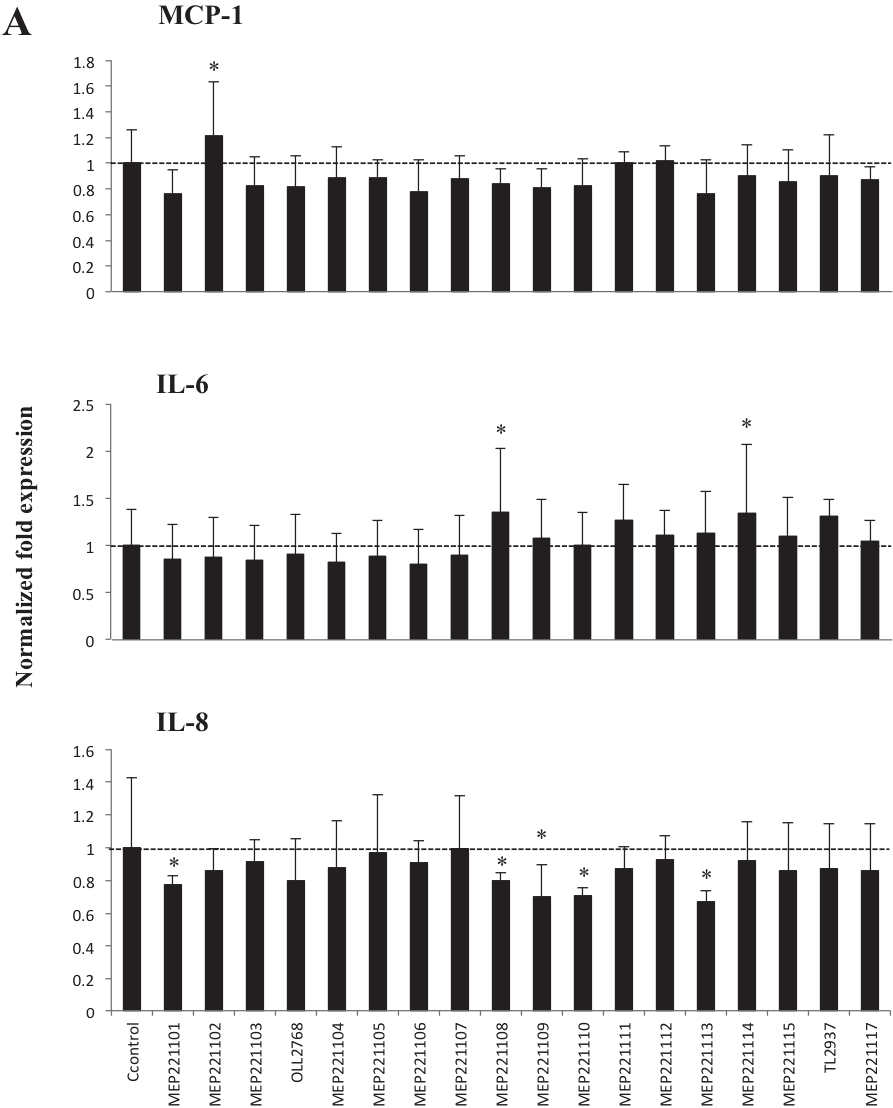

**B**

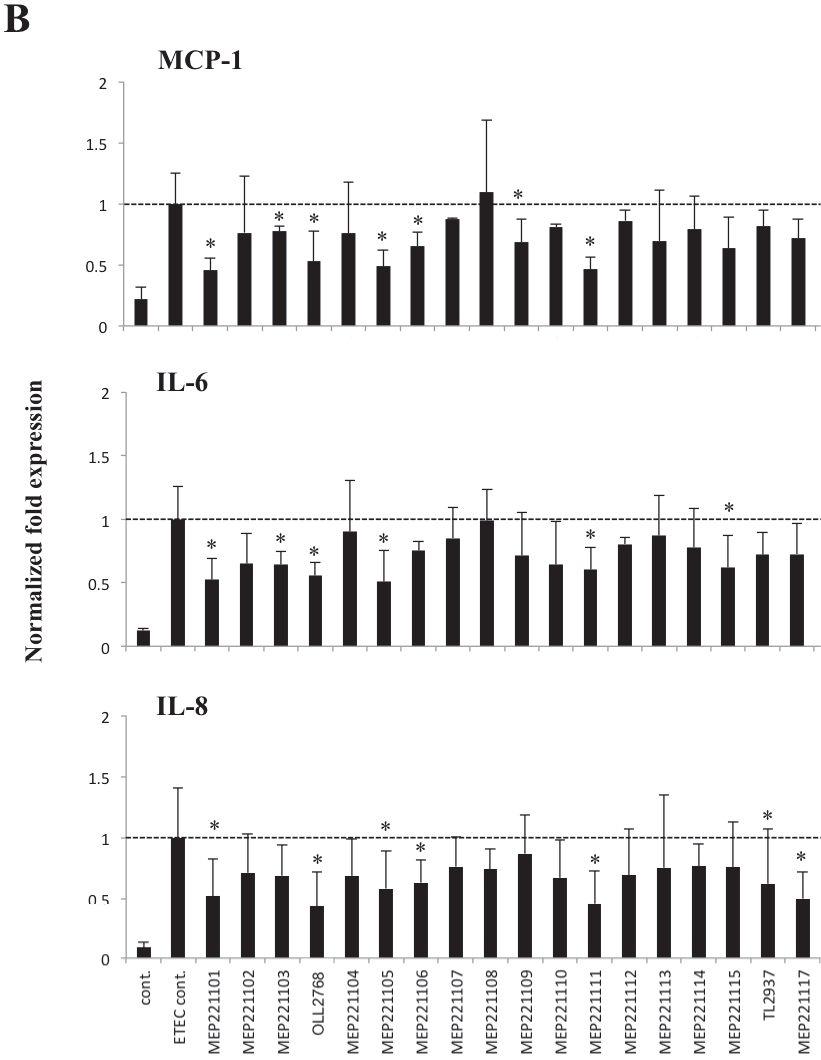

Supplement: Additional file 1: Figure S1 — Selection of immunomodulatory lactobacilli. (A) BIE cells were pre-treated with different lactobacilli strains for 48 hours and the expression of MCP-1, IL-6 and IL-8 was studied. Values represent means and error bars indicate the standard deviations. The results represent five independent experiments. Significantly different from control *(P<0.05). (B) BIE cells were pre-treated with different lactobacilli strains for 48 hours and the stimulated with heat-stable ETEC PAMPs and then the expression of MCP-1, IL-6 and IL-8 was studied at hour twelve post-stimulation. Values represent means and error bars indicate the standard deviations. The results represent five independent experiments. Significantly different from ETEC control *(P<0.05). [file 1471-2180-13-54-S1.pdf]
